# Gallation and B-Ring Dihydroxylation Increase Green Tea Catechin Residence Time in Plasma by Differentially Affecting Tissue-Specific Trafficking: Compartmental Model of Catechin Kinetics in Healthy Adults

**DOI:** 10.3390/nu15184021

**Published:** 2023-09-17

**Authors:** Joanna K. Hodges, Geoffrey Y. Sasaki, Yael Vodovotz, Richard S. Bruno

**Affiliations:** 1 Human Nutrition Program, The Ohio State University, Columbus, OH 43210, USA; 2 Department of Nutritional Sciences, The Pennsylvania State University, University Park, PA 16802, USA; 3 Department of Food Science and Technology, The Ohio State University, Columbus, OH 43210, USA

**Keywords:** bioavailability, catechins, green tea, human, mathematical modeling, metabolism

## Abstract

Catechins in green tea extract (GTE) (epigallocatechin gallate (EGCG), epigallocatechin (EGC), epicatechin (EC), epicatechin gallate (ECG)) vary in bioactivity. We developed a physiologically relevant mathematical model of catechin metabolism to test the hypothesis that fractional catabolic rates of catechins would be differentially affected by their structural attributes. Pharmacokinetic data of plasma and urine catechin concentrations were used from healthy adults (*n* = 19) who ingested confections containing 0.5 g GTE (290 mg EGCG, 87 mg EGC, 39 mg EC, 28 mg ECG). A 7-compartmental model of catechin metabolism comprised of the gastrointestinal tract (stomach, small and large intestine), liver, plasma, extravascular tissues, and kidneys was developed using a mean fraction dose of EGCG, ECG, EGC, and EC. Fitting was by iterative least squares regression analysis, and goodness of fit was ascertained by the estimated variability of parameters (FSD < 0.5). The interaction of gallation and B-ring dihydroxylation most greatly extended plasma residence time such that EGC > EC = EGCG > EGC. The interaction between gallation and B-ring dihydroxylation accelerated the transfer from the upper gastrointestinal tract to the small intestine but delayed subsequent transfers from the small intestine through the liver to plasma and from kidneys to urine. Gallation and B-ring dihydroxylation independently delayed the transfer from plasma to extravascular tissues, except the uptake to kidneys, which was slowed by gallation only. This multi-compartment model, to be validated in a future study, suggests that gallation and B-ring dihydroxylation affect catechin catabolism in a tissue-specific manner and thus their potential bioactivity.

## 1. Introduction

**Figure 1:**
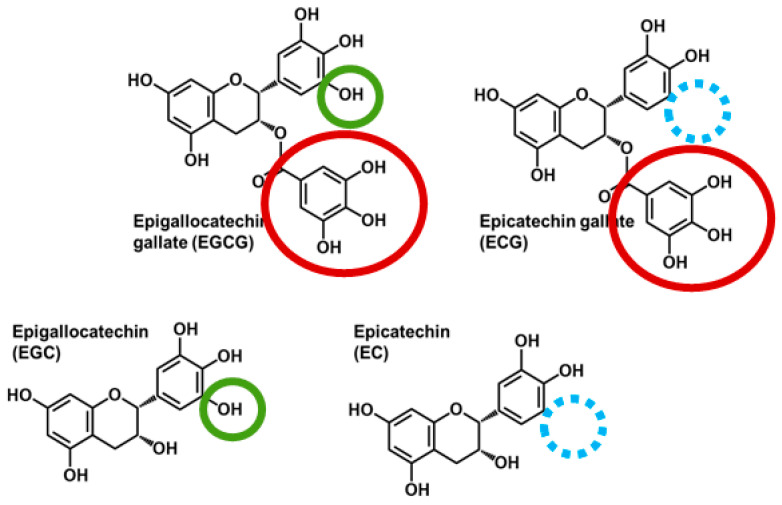
Chemical structures of major catechins present in green tea. Epigallocatechin gallate (EGCG) and epicatechin gallate (ECG) contain a gallate group (red circle), whereas EGCG and epigallocatechin (EGC) are trihydroxylated on the B-ring (green circle) compared with ECG and epicatechin (EC) that are dihydroxylated (blue circle indicates the site of the missing hydroxyl group).

Observational studies, human trials, and/or controlled studies in preclinical models support that green tea consumption reduces the risk of cardiovascular disease [1,2], certain cancers [3], and cardiometabolic disorders including obesity [4,5], type 2 diabetes mellitus [6,7,8,9], and nonalcoholic fatty liver disease [10,11]. Its health-promoting activities are attributed to its high catechin content, specifically epigallocatechin gallate (EGCG), epicatechin gallate (ECG), epigallocatechin (EGC), and epicatechin (EC; Figure 1). The catechins differ structurally based on the presence of a gallate group (i.e., EGCG and ECG) and the number of hydroxyl groups on the B ring. However, limited information exists about the influence of catechin structure on their absorption, transfer between biological pools, and elimination, which are important for a better understanding of their compound-specific health-promoting bioactivities.

EGCG is regarded as the major bioactive component of green tea due to its high abundance (50–75% of total catechin content [12]) and superior free radical scavenging activity in vitro [13]. Paradoxically, although all catechins are poorly bioavailable, EGCG is the least bioavailable of the catechins [14]. For example, only 0.16% of EGCG following oral administration of green tea was present in human plasma compared with 0.58% and 1.1% of EGC and EC, respectively [14]. Low EGCG bioavailability is attributed to its high efflux from enterocytes back into the intestinal lumen by the multidrug resistance-associated proteins and P-glycoprotein [15,16]. Contrary to what could be inferred from its low bioavailability, studies in a Caco-2 monolayer demonstrated that EGCG had a lower degree of efflux compared with the non-gallated catechins (EC and EGC) [16]. Because disparity exists between catechin bioavailability and bioactivity, as exemplified by EGCG [14], more research is needed to elucidate the fate of catechins beyond their absorptive process. Alternative quantitative approaches are also expected to further an understanding of how structural attributes of catechins affect their trafficking and, thus, their biological activity.

Evidence suggests that catechin half-lives are influenced by their structural attributes. For example, the gallate group of EGCG favors its binding to proteins, including serum albumin [17,18]. This prevents EGCG from crossing the renal glomerular filtration barrier to limit its urinary excretion [19,20], thus extending its half-life and potential for bioactivity at target tissues [14,21]. However, whether protein binding influences the residence time bioactivity of other catechins is unknown. Thus, non-invasive techniques that can evaluate tissue-specific uptake and efflux of catechins will help to define their structure/function relationships on health outcomes. Model-based compartmental analysis can quantitatively assess catechin metabolism in a compound- and organ-specific manner to clarify the paradox between catechin bioavailability and bioactivity. In compartmental analysis, each compartment represents a pool of a compound within a physiological region (e.g., liver), where the compound is expected to behave in a kinetically uniform manner. Uptake and efflux of catechins between compartments can be estimated by fractional transfer coefficients, which represent the fraction of compound transferred or lost irreversibly from a compartment per unit of time. Further, rather than using the catechin concentration in plasma, which depends on the dose, compartmental modeling normalizes data to dose to enable systematic comparisons between analytes’ modeled parameters.

The main objective of this study was to develop a compartmental model of catechin metabolism in humans to calculate and compare the fractional transfer of individual catechins between plasma and organs to better understand their tissue-specific metabolism relative to their structural attributes. Based on pharmacokinetic studies in healthy adults showing that EGCG had a longer elimination half-life than EGC and EC [14], we hypothesized that gallated catechins (EGCG and ECG) would have a longer plasma residence time compared with non-gallated EGC and EC due to slower transfer from plasma to kidneys. We further hypothesized that catechins having a dihydroxylated B-ring would be less rapidly depleted, consistent with their lower potential for free radical scavenging [22,23]. To test these hypotheses, we utilized data from a human pharmacokinetic study that examined the bioavailability and urinary elimination of catechins [24]. The outcomes of the present study are, therefore, expected to advance an understanding of the efficacy of green tea and its catechins in managing disease risk.

## 2. Materials and Methods

### 2.1. Participants

Data for the present study were obtained from our prior investigation of the pharmacokinetics of green tea polyphenols in healthy adults [24]. The study protocol was approved by the Biomedical Institutional Review Board at Ohio State University (#2017H0246) and registered at ClinicalTrials.gov (NCT03413735). In brief, 19 healthy adults (10 males and 9 females; 27 ± 2 y; 22 ± 0 kg/m^2^) who were normotensive (systolic blood pressure, 112.1 ± 2.3 mmHg and diastolic blood pressure, 72.5 ± 1.6 mmHg) normoglycemic (fasting glucose, 92.3 ± 1.8 mg/dL) and normolipidemic (total cholesterol, 181.1 ± 7.8 mg/dL and triglyceride 61.7 ± 7.5 mg/dL) completed the study in the Columbus, Ohio area. Participants were non-smokers, non-users of dietary supplements, non-regular tea drinkers (<2 cups/wk), consumed <3 alcoholic drinks/d, non-pregnant or non-lactating, not using any medications or antibiotics, and had no history of gastrointestinal disorders or surgeries.

### 2.2. Study Design

**Figure 2:**
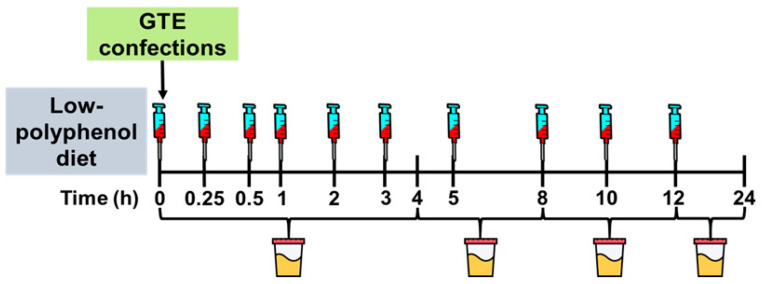
Study Design. Healthy adults completed a pharmacokinetics study in which they ingested a gelatin-based confection containing green tea extract (445 mg total catechins; 290 mg epigallocatechin gallate, 87 mg epigallocatechin, 39 mg epicatechin, 28 mg epicatechin gallate). Blood samples were collected at timed intervals for 12 h and urine was collected for 24 h. Analyzed catechins were used to construct a multi-compartment model of tissue and plasma transfer of catechins.

Complete details of the pharmacokinetic study were reported in [24]. In brief, participants abstained from polyphenol-rich foods for 3 d. In the fasting state (10–12 h) on day 4, participants voided their bladder, and a catheter was inserted into the antecubital vein to collect blood samples at baseline (0 h) and at 0.25, 0.5, 1, 2, 3, 5, 8, 10, and 12 h post-ingestion of a confection formulated with catechin-rich GTE. Urine was collected in timed intervals from 0–4, 4–8, 8–12, and 12–24 h (Figure 2). Participants received standardized meals at 4 h (11:00), 6 h (13:00) and 12 h (19:00) following confection ingestion. Snacks (e.g., pretzels, crackers) were also provided between 13:00–19:00. All foods were devoid of polyphenols and provided to participants in a eucaloric manner based on estimated energy requirements using the Harris–Benedict formula [25]. Controlled meals were designed to provide 48–55% of energy from carbohydrates, 15–20% from proteins, and 20–35% from fats. No additional foods or beverages, except water, were consumed by participants during the 24 h trial period.

### 2.3. GTE Confections

GTE confections were developed as an alternative delivery system for catechins because Americans prefer catechin-deplete black tea over catechin-rich green tea [26]. Confections were prepared in-house and formulated with 84.5% water, 2% sucrose, 6% gelatin, 0.5% citric acid, 6% lime-flavored gelatin, and 1% decaffeinated GTE powder. Confections were prepared within 24 h of the participants’ pharmacokinetics trial and stored at 4 °C. Each participant ingested 2 confections (each 25 g and ~2.5″ cubed), which contained 0.5 g GTE (Sunphenon 90 LB, Taiyo International, Inc., Minneapolis, MN, USA) to provide 445 mg total catechins (290 mg EGCG, 87 mg EGC, 39 mg EC, 28 mg ECG). The catechin content of all confections was confirmed by HPLC analysis [27]. Based on brewed green tea (250 mL prepared with 2.2 g of dry tea leaves) containing ~180 mg total catechins [28], GTE at 0.5 g approximates 2.5 cups of green tea.

### 2.4. Biospecimen Preservation and Catechin Analysis in Plasma and Urine

Biospecimen handling and preservation of catechins concentrations in plasma and urine were described [10]. In brief, venous plasma was collected in heparinized tubes, while urine was collected in sterilized containers. Samples were preserved by adding a solution of acetic acid, ascorbic acid, and EDTA, snap-frozen in liquid nitrogen and stored at −80 °C until analyzed. Plasma and urinary catechins (EGCG, EGC, ECG, EC) were analyzed by LC-MS following enzymatic hydrolysis and extraction as reported [24].

### 2.5. Compartmental Model Development and Kinetic Analysis

Tissue-specific kinetics of each catechin were determined by model-based compartmental analysis using the Windows version of Simulation, Analysis, and Modeling software (WinSAAM version 3.0.8) [29]. The observed data were expressed as a fraction of the ingested dose, i.e., the mass of catechins measured in plasma/urine at specified times divided by the mass of the ingested catechin dose. The mass of catechins in participants’ plasma was derived from their plasma concentration multiplied by their estimated plasma volume calculated from height and weight as detailed [30]. Urinary catechin concentrations were multiplied by the total urine volume collected at each collection interval to calculate the mass of excreted urinary catechin.

The initial conceptualization of compartmental model structure (number of compartments and their connections) was informed by published studies of catechin metabolism in humans [31,32,33,34]. Fractional absorption was defined as the fraction of catechin moving from the small intestine to the liver and initially set at 30% per hour based on a prior report [34] and subsequently made adjustable to account for the known large inter-individual variation in catechin bioavailability [31]. The ratio of fractional absorption to fractional excretion in feces was fixed at 1:2 to allow for more degrees of freedom when estimating the fractional transfers past absorption and between plasma and tissues, which is the focus of this study. To test the model structure against observed kinetic data, the mean fraction of dose of the sum of all four catechins (EGCG, EGC, ECG, and EC) recovered from plasma was plotted over time and compared against the simulated plot generated by WinSAAM. The model structure was iteratively adjusted until a close fit was obtained, as judged by visual inspection of the simulated plot against the observed data, the sum of squares from nonlinear regression analysis, and the estimated variability (fractional standard deviation) of each kinetic parameter. Model complexity (and thus the number of parameters) was increased only when it improved the sum of squares and the estimated variability of parameters.

Once a satisfactory fit was achieved of the model simulation to the mean total catechin data, data from individual participants were used to model the kinetics of the 4 catechins separately. Fractional transfer coefficients (the fractions of catechin transferred from one compartment to another per h) denoted as L(I,J)s with J representing the origin compartment and I representing the destination compartment were adjusted in a stepwise manner to obtain the best fit of the model-simulated plot to the observed data. After a satisfactory fit was achieved, the final values of the transfer coefficients were generated through a weighted nonlinear regression analysis, which minimized the residuals given the weight assigned to the observed data, parameter constraints, and their uniqueness. The parameters were considered well identified if the sum of squares from regression analysis was <10^−5^, the estimated error of parameters was <0.5 (i.e., the estimated error could not exceed 50% of the parameter value), and the correlation between parameters was <0.8 (i.e., each parameter described a discrete transfer rate without redundancy in parameters). Using the fractional transfer coefficients obtained from the model, the following two kinetic parameters were calculated: residence time in plasma, i.e., the mean of the distribution of times a catechin spends in plasma before irreversible disposal (calculated from the L-inverse matrix in WinSAAM), and fractional catabolic rate (FCR), i.e., the fraction of catechin lost irreversibly from plasma per min (calculated from the reciprocal of residence time).

### 2.6. Statistical Analysis

All data (means ± SEMs) were analyzed using GraphPad Prism for Windows (version 9.1), GraphPad Software (Boston, MA, USA). We initially explored the effects of sex, gallation, and hydroxylation using a three-way ANOVA. Although the mean FCR was 15% (13% EGC, 33% EGCG, 9% EC, 5% ECG) higher in females compared with males, this occurred without any statistically significant effect of gender on FCR (the primary outcome) or any other kinetic parameter. Thus, all subsequent analyses were conducted using a two-way ANOVA with Tukey’s post-hoc analysis when appropriate to assess the effect of gallation, degree of hydroxylation on B-ring, and their interaction. *p* ≤ 0.05 was considered statistically significant.

## 3. Results

### 3.1. Model Structure

**Figure 3:**
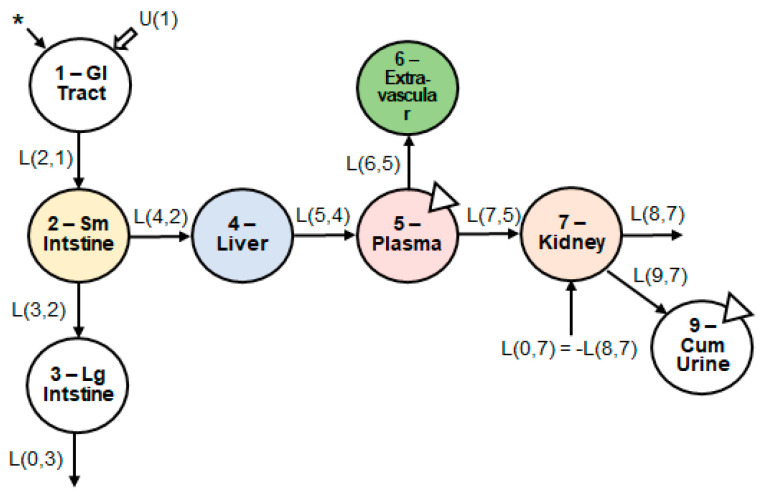
Structure of the multi-compartment model. Seven body compartments, including the upper GI tract, small and large intestine, liver, plasma, extravascular tissues, and kidneys, were required to obtain the best model fit to the observed data. The asterisk indicates the site of catechin introduction to the physiological system. U(1) indicates any introduction of dietary catechins, which was negligible due to dietary restriction. The triangles indicate the sites of biospecimen sampling. Abbreviations: GI, gastrointestinal; Sm, small; Lg, large; Cum, cumulative.

The initial model structure consisted of six compartments: (1) upper gastrointestinal tract; (2) small intestine (i.e., site of absorption); (3) large intestine; (4) liver; (5) plasma; (6) kidney. We tested the following iterations of the model: adding a compartment representing the portal vein, adding excretion via bile, adding enterohepatic circulation, and adding a compartment representing the extravascular tissues (e.g., muscle, skin, brain). None of the iterations, except for adding an extravascular tissue compartment, improved the fit of the model to the data. The final model structure comprised of seven compartments (Figure 3).

### 3.2. Response Profiles of Catechins in Plasma and Urine

**Figure 4:**
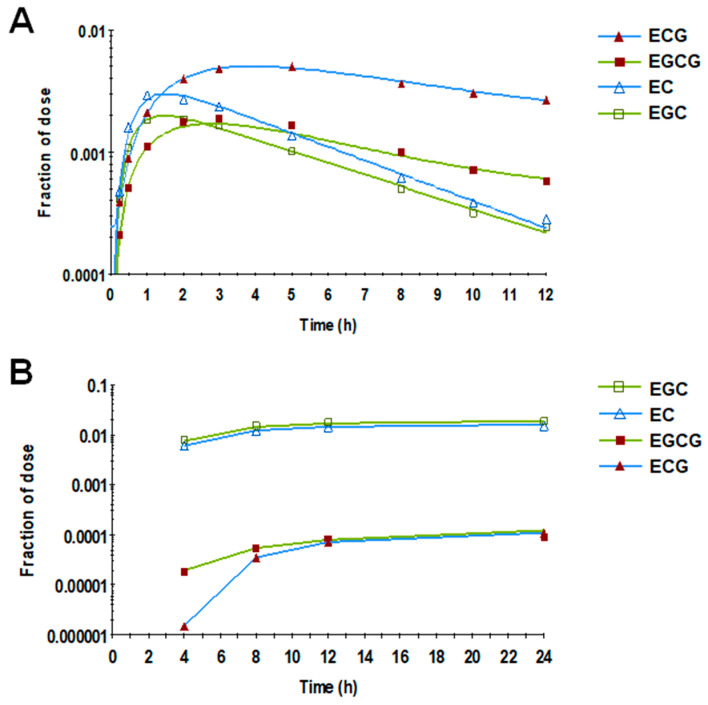
Mean fraction of the catechin dose in plasma (**A**) and urine (**B**) over time. Gallated catechins showed less steep terminal slopes in plasma and a lower fraction of dose in urine compared with the non-gallated catechins. Symbols represent the observed data (*n* = 19), and lines represent the model prediction.

Based on visual inspection of the response profile of the four individual catechins in plasma (Figure 4A), the terminal elimination slopes of the two gallated catechins (EGCG and ECG) were less steep than those of the non-gallated ones (EGC and EC), suggesting a relatively slower rate of elimination of the gallated catechins. In agreement, the non-gallated catechins showed, on average, a 150-fold higher fraction of dose eliminated in urine compared with gallated catechins (Figure 4B).

### 3.3. Fractional Catabolic Rate and Residence Time

**Figure 5:**
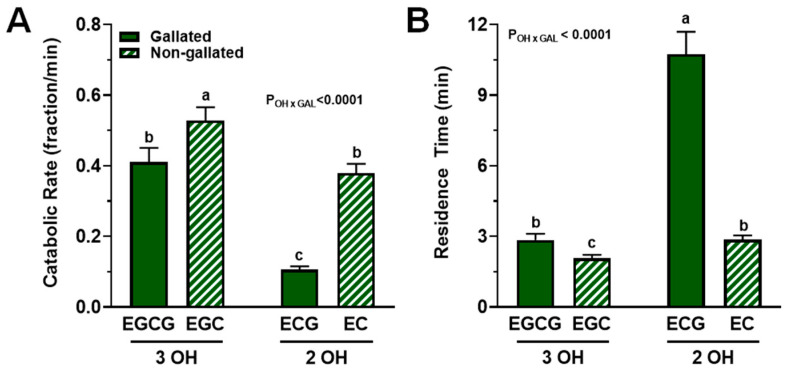
FCR and residence times of catechins in plasma from healthy adults who ingested a green tea extract-containing confection. (**A**) Catechin FCR was affected by an interaction between the degree of hydroxylation and the presence of a gallate group. While trihydroxylated and non-gallated catechins had greater FCR, their structural combination potentiated the FCR such that ECG > EGCG = EC > ECG. (**B**) The degree of hydroxylation and presence of a gallate group interacted significantly such that dihydroxylation and gallation most greatly increased residence time of catechins: ECG > EC = EGCG > EGC. Data (means ± SE, *n* = 19 subjects) were analyzed by two-way ANOVA with Tukey’s post-hoc test. Group means not sharing a common letter are different from each other (*p* < 0.05) such that a > b > c. Abbreviations: EC, epicatechin; ECG, epicatechin gallate; EGC, epigallocatechin; EGCG, epigallocatechin gallate; FCR, fractional catabolic rate; GAL, gallated; OH, degree of hydroxylation.

The fractional catabolic rate (FCR), i.e., the fraction of compound lost irreversibly from plasma per min, was significantly influenced by structural attributes of catechins (*P_interaction_* < 0.0001; Figure 5A). While trihydroxylated catechins (EGCG and EGC) had, on average, a 48% greater FCR compared with dihydroxylated catechins (ECG and EC) and those catechins devoid of a gallate group had a 43% greater FCR (EGC and EC vs. EGCG and ECG), a statistically significant interaction between these structural attributed was observed (*p* < 0.001). Indeed, the FCR of catechins was potentiated by the combination of trihydroxylation and the absence of gallation (EGC > EGCG = EC > ECG; Figure 5A).

A similar but inverse trend was observed for plasma residence time, i.e., the mean of the distribution of times catechin spend in plasma after entering via the liver before irreversible loss (*P_interaction_ <* 0.0001; Figure 5B). Data indicated that the combination of dihydroxylation and gallation most greatly increased plasma residence time: EGC > EGCG = EC > ECG.

### 3.4. Tissue-Level Kinetics

**Figure 6:**
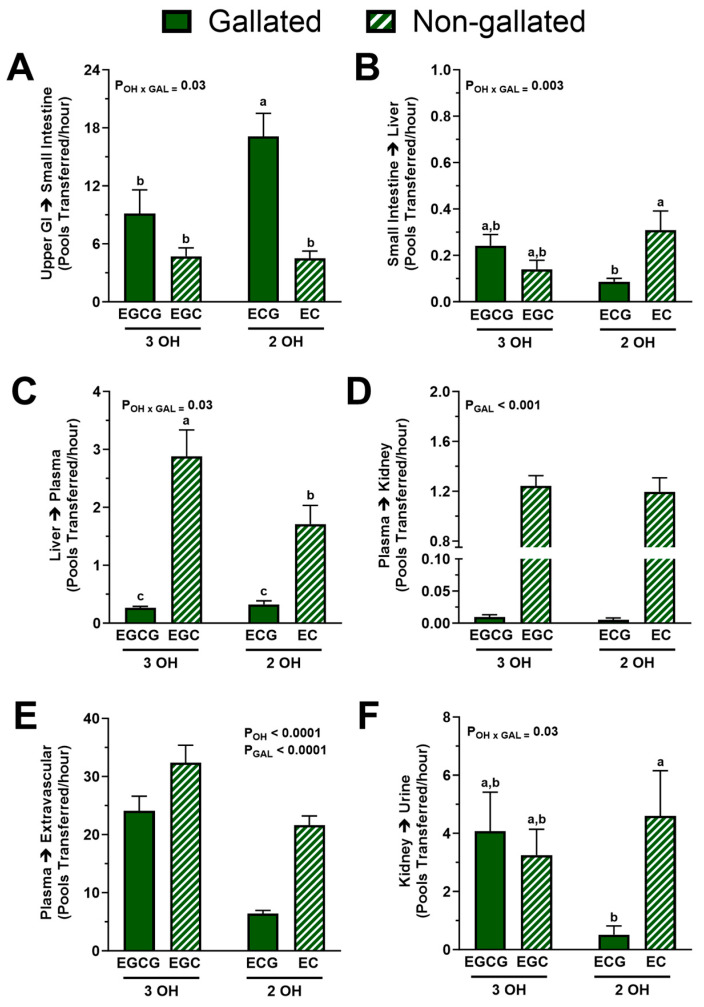
Fractional transfer rates of catechins between body pools from healthy adults who ingested a green tea extract-containing confection. (**A**) The degree of hydroxylation and presence of a gallate group interacted such that dihydroxylated and gallated ECG had a higher fractional transfer rate from upper GI tract to small intestine. (**B**) The degree of hydroxylation and presence of a gallate group interacted such that gallation slowed down the fractional transfer from small intestine to liver but only for the dihydroxylated catechin ECG. (**C**) The degree of hydroxylation and presence of a gallate group interacted such that combination of gallation and B-ring dihydroxylation most greatly attenuated the fractional transfer rate of catechins from liver to plasma: EGC > EC > EGCG = ECG. (**D**) Presence of a gallate group, but not hydroxylation, decreased the fractional transfer rate from plasma to kidneys: EGC = EC > EGCG = ECG. (**E**) Gallation and dihydroxylation independently decreased the fractional transfer rate from plasma to extravascular tissues: EGC > EGCG > EC > ECG. (**F**) The effect of hydroxylation and gallation on the fractional transfer from kidneys to urine was identical to that found on the transfer from small intestine to liver. Data (means ± SE, *n* = 19 subjects) were analyzed by two-way ANOVA with Tukey’s post-hoc test. Group means not sharing a common letter are different from each other (*p* < 0.05) such that a > b > c. Abbreviations: EC, epicatechin; ECG, epicatechin gallate; EGC, epigallocatechin; EGCG, epigallocatechin gallate; FCR, fractional catabolic rate; GAL, gallated; OH, degree of hydroxylation.

We next investigated whether structural attributes of green tea catechins influenced their fractional transfer rates between compartments. A significant interactive effect of gallation and degree of hydroxylation was found in the fractional transfer rate through the upper gastrointestinal tract to the liver. Post-hoc analysis indicated that the fractional transfer rate from the upper gastrointestinal tract to the small intestine was faster for ECG than for other catechins, which showed no significant between-compound differences (Figure 6A), whereas the transfer from the small intestine to the liver was faster in the absence of gallation only among dihydroxylated ECG and EC (Figure 6B).

For the fractional transfer rate of catechins from the liver to plasma, we observed a statistically significant interaction between the degree of hydroxylation and gallation (*p* < 0.05). The combination of trihydroxylation and the lack of gallation potentiated catechin transfer from the liver to plasma such that EGC > EC > EGCG = ECG (Figure 6C). However, absence of a gallate group, but not the degree of hydroxylation, increased the fractional transfer rate from plasma to kidneys (EGC = EC > EGCG = ECG; *p* < 0.0001; Figure 6D). Indeed, the non-gallated catechins had a 150-fold faster uptake into the kidneys compared with those that are gallated. Further, trihydroxylation (*p* < 0.001) and the absence of gallation (*p* < 0.001) but not their interaction increased the uptake of catechins by other extravascular tissues (Figure 6E). Lastly, the fractional transfer from kidneys to urine showed the same pattern as the transfer from the small intestine to the liver (Figure 6F), i.e., it was higher in the absence of the gallate group for the B-ring dihydroxylated catechins.

## 4. Discussion

This investigation has established a multi-compartmental mathematical model to understand how structural attributes of green tea catechins, specifically their degree of hydroxylation on the B-ring and the presence of a gallate group, influence their trafficking between organ systems in healthy adults. Data show, consistent with our hypothesis, that the combination of gallation and dihydroxylation most greatly prolongs catechin residence time in plasma (i.e., decreases fractional catabolic rate), likely by reducing irreversible uptake into the kidneys. Further, catechin gallation and dihydroxylation likely contribute to extended plasma residence time by reducing catechin depletion in extravascular tissues. These outcomes advance the existing knowledge of catechin bioavailability [24] and pharmacokinetics [14] by providing critical insight into catechin trafficking from the intestine to the liver and other extrahepatic tissues without the need for highly invasive tissue biopsy, which is a unique advantage of model-based compartmental analysis. Indeed, tissue-specific kinetics of catechins can better predict their bioactivity compared with bioavailability alone [32], thus providing a unique foundation for designing catechin-specific interventions to target health disorders of differing organ systems.

Evidence from numerous epidemiological studies suggests that the greater intake of green tea affords protection against diseases affecting multiple organs and organ systems, including the cardiovascular system, liver, gastrointestinal tract, adipose tissue, and bones and teeth [35]. Recent in vitro and preclinical studies also demonstrate differential bioactivities of individual catechins depending on the study endpoint [10,36,37,38,39,40]. For example, studies in vitro demonstrate the superior free radical scavenging activity of EGCG [13] followed by ECG, EGC, and EC [22], indicating that catechin bioactivity is predicted by the number of hydroxyl groups present [41]. In contrast, anti-inflammatory activity was superior for ECG and catechin gallate compared with EGCG in pancreatic tumor cells [42]. These findings indicate that it is insufficient to consider only the intakes of green tea catechins and that an understanding of catechin-specific metabolism and bioactivities is needed to improve human health.

Bioavailability and pharmacokinetic studies in humans are routinely performed, including by our group [24], because they can help to explain the potential bioactivities of green tea catechins. However, these studies have limitations because biospecimens are often only collected from blood and/or urine rather than tissues where bioactivities would be expected. Further, in studies examining catechins from green tea or GTE, the doses of individual catechins are different from each other, thereby limiting an understanding of catechin-specific bioactivities. To this end, a study by Dey et al. [10] was unique in design because it directly compared the anti-inflammatory activities of purified EGCG and (+)-catechin, a stereoisomer of EC, following their supplementation at equal doses in diets of obese mice. Their findings demonstrated that catechin was effective in alleviating pathological evidence of nonalcoholic steatohepatitis, but EGCG was more potent [10]. This is consistent with our findings showing that the influx of EGCG and EC into the liver was similar but that the hepatic efflux of EGCG was slower compared with EC (Figure 6C). This may indicate an enhanced propensity of the liver to store EGCG (e.g., as bound to proteins) and, thus, potentially longer opportunity for EGCG to exert its anti-inflammatory activity. Further, our evidence shows that prior to the efflux from the liver, there was no difference between EGCG and EC in their gastrointestinal trafficking. This could explain the identical potency of EGCG and (+)-catechin to maintain the expression of intestinal tight junction proteins and alleviate intestinal inflammation [10]. A better characterization of the different fates of catechins at different organs and organ systems, as shown herein with the use of compartmental analysis, may explain their tissue-specific bioactivities and provide a foundation for catechin-specific interventions depending on the organ system that will be targeted.

We observed that plasma catechin residence time, which reflects the net elimination rate versus circulation in plasma, was extended by the interaction between gallation and dihydroxylation on the B-ring (Figure 5). The former is consistent with the gallate group enhancing catechin-albumin binding [20,43]; albumin is the most abundant plasma protein, accounting for >50% of all plasma proteins [44,45]. Binding to albumin likely protects gallated catechins from oxidation by free radicals, thereby extending their residence time in plasma. Gallated catechins are also less likely to be conjugated and thus less likely to be eliminated in the urine. Indeed, gallated catechins are present in plasma mainly as unconjugated forms, whereas non-gallated catechins are usually conjugated [14,46], a xenobiotic process that facilitates their elimination and which is consistent with the relatively lower fraction of dose in the urine of the gallated catechins compared with the non-gallated ones (Figure 4B). The interaction of B-ring dihydroxylation with gallation leading to even longer plasma residence time (Figure 5B) is also consistent with our hypothesis that dihydroxylated catechins are less likely to be depleted in line with their reported activities of partaking in less free radical scavenging. Indeed, in the absence of binding to albumin, the relative capacity for free radical scavenging in vitro is lower for EC compared with EGC and lower for ECG compared with EGCG [22,23]. This implies that catechins having fewer un-bound hydroxyl groups on the B-ring are less susceptible to oxidative depletion.

We next investigated how structural attributes of catechins affected their kinetics in specific tissues. Unlike plasma residence time, catechin efflux from the liver to plasma was primarily affected by gallation (Figure 6C), as was the uptake by the kidneys, which showed an exclusive gallation effect (Figure 6D). This could be explained by the binding to albumin, which protects catechins from renal filtration. The proportion of albumin that undergoes glomerular filtration is relatively low (0.1–4% [45]) due to its large size and low isoelectric point [44,47], which may shield albumin-bound catechins from renal uptake. Binding to albumin may also explain the slower efflux of gallated catechins from the liver, where albumin is synthesized and stored [45]. Consistent with albumin limiting renal elimination of catechins, the uptake of catechins to extravascular tissues from plasma was also hindered by gallation (Figure 6E). Notably, 60–70% of the total body albumin is found in the interstitial fluid of skin and muscle [45]. Binding to albumin may prevent catechins from being sequestered by the extravascular tissue cells by trapping them in the interstitial space and allowing them to re-enter the plasma. However, unlike the uptake into kidneys, the uptake into other extravascular sites also showed a pronounced and independent effect of B-ring hydroxylation such that, consistent with our hypothesis, the dihydroxylated catechins were slower to be taken up and/or be lost (Figure 6E). This may be due to a much larger surface area of all the extravascular sites compared with the liver and kidneys and, thus, a greater propensity of the catechins to interact and be depleted by free radicals.

At the gastrointestinal tract, ECG showed relatively faster transit until the small intestines, where the EC was then favored over EGC for trafficking to the liver, suggesting that the gallate group impedes catechin bioavailability (Figure 6A,B). The acidic environment of the stomach helps to protect catechins from auto-oxidation with no notable differences between specific catechins [48,49]. Conversely, the alkaline environment of the small intestine promotes catechin degradation, with relatively greater loss of EGCG and EGC compared with ECG and EC [49,50]. Further, studies investigating intestinal transport of catechins in a Caco-2 cell model showed basal-to-apical transport of catechins in the following order: EC > EGC > ECG ≈ EGCG [16,46]. This suggests that the gallated catechins, ECG and EGCG, have more limited absorption potential due to their greater preference to interact with efflux transporters (multidrug resistance proteins) [31]. This concept is consistent with our observation that ECG trafficking from the intestine to the liver is slower than that of EC (Figure 6B). The slower transit to the liver of ECG compared with EC could be attributed to its greater oxidative depletion [22], which could also explain the slower transfer of ECG vs. EC from the kidney to urine (Figure 6F).

Strengths of this study include the use of compartmental modeling, which captures the continuum of catechin trafficking without having to collect biospecimens at impractically short and frequent time intervals. This is particularly useful for polar compounds, such as catechins, that exhibit rapid absorption and elimination relative to lipophilic compounds that are more readily stored. Another strength is the ability to estimate and compare parameters of catechins on a per-dose basis (i.e., rates are expressed as a fraction of the ingested dose over time). This is important because the proportion of catechins varies within GTE. Normalizing parameters to the dose ingested permits direct comparison among transfer rates without having to administer catechins in equal quantities. Compartmental modeling also estimates the uptake and efflux of catechins in and out of plasma and organ systems, thus providing a tissue-specific representation of catechin metabolism in a physiologically relevant manner. Limitations of this study include the possibility that catechins interact with each other and/or interconvert during microbial metabolism [51]. Our parameter estimates also depend on the type and masses of catechins recovered from plasma. For example, if a substantial portion of the gallated catechins (EGCG and ECG) are hydrolyzed to their corresponding non-gallated forms (EGC and EC), parameter estimates would be skewed. However, this is unlikely based on a study in healthy humans that provided equal amounts (1.5 mmol) of ECG, EGC, or EGCG in a crossover design showing that de-gallation and interconversion of catechins (ECG → EC, EGCG → EGC) is very limited [52]. Another limitation is the possibility that tissue uptake, especially during absorption, is saturated, which could underestimate the fractional transfer of catechins. Finally, our model does not permit an estimation of the specific proportion of catechins that are taken up or lost in compartments, such as the extravascular tissues, nor does it differentially assess the kinetics of conjugated vs. unconjugated catechins. Future preclinical studies that quantify catechin catabolism, including their oxidation and conjugation, at various organs are needed to further explain their tissue-specific bioactivities.

## 5. Conclusions

Compartment modeling is an important quantitative tool that can expand the understanding of catechin trafficking and the physiological influence of their structural attributes on between-tissues transfer, which provides insight into their tissue-specific bioactivities. Here, we show that the combination of gallation and dihydroxylation on the B-ring most greatly prolongs catechin residence time in plasma, likely by reducing irreversible uptake into the kidneys and depletion at other extravascular tissues. While these outcomes help to explain tissue-specific bioactivities of catechins and provide the foundation for catechin- and organ-specific interventions, future studies are needed to identify the mechanisms by which catechin metabolism is differently regulated at different tissues and how the gut microbiota, which is known to catabolize catechins [51] influences the metabolism and bioactivity of catechins.

## Data Availability

Data are available from the study authors upon reasonable request.
